# Acknowledgement to Reviewers of *Children *in 2017

**DOI:** 10.3390/children5020015

**Published:** 2018-01-23

**Authors:** Children Editorial Office

**Affiliations:** MDPI AG, St. Alban-Anlage 66, 4052 Basel, Switzerland

Peer review is an essential part in the publication process, ensuring that *Children *maintains high quality standards for its published papers. In 2017, a total of 115 papers were published in the journal. Thanks to the cooperation of our reviewers, the median time to first decision was 28 days and the median time to publication was 57 days. The editors would like to express their sincere gratitude to the following reviewers for their time and dedication in 2017:
Abramo, Thomas J.Lopez, NanetteAgarwal, AmitLucendo, AlfredoAlaofe, HalimatouLucey, AliceAllegaert, KarelMa, Zheng FeeiAllen, TarynMaclellan, Reid A.Amini, Rose MarieMahieu, LudoArca, MarcelloMalby Schoos, Ann-MarieArdigò, LucaMandrell, BelindaArnbjörnsson, EinarMars, MauriceAsakura, KeikoMartin, StephanieAugust, Keith J.Maslin, KateAyán Pérez, CarlosMassotte, DominiqueBaker, EmmaMattsson, JanetBaker, Mark DouglasMayer, AntonBarbi, Egidio A.McDonnell, AndrewBarnes, GregoryMcIntosh, E. David G.Barone, Daniel A.McIntosh, IanBarton, KarenMcKenna, Malachi J.Beck, Suzanne E.McLoney, Eric D.Becker, DavidMearelli, FilippoBender, Hans-UlrichMetzger, Monika L.Berens, Pamela D.Meyer, Walter J.Berezin, EitanMignon, SylviaBerger, RolfMisra, Sanghamitra M.Bergstraesser, EvaMoise, Imelda K.Beslow, Lauren A.Monk, Ellis P.Bhandari, VineetMorrison, Wynne E.Bhat, SushanthMuñoz-López, FranciscoBiank, Vincent F.Munshi, AdityaBisulli, FrancescaMusher-Eizenman, Dara R.Blackall, Douglas P.Mutiga, Samuel K.Blankestijn, MarkNaim, MitreBorden, LynneNakai, KunihikoBoshuizen, Henny P.A.Natale, VincenzoBoss, ReneeNazare, BarbaraBravaccio, CarmelaNewby, RuthBremer, Andrew A.Newmark, SanfordBrennan, PatriciaNeymotin, FlorenceBreuner, Cora ColletteNobmann, Elizabeth D.Brierley, DanielNoel, MelanieBrilli, RichardNoonan, VikkiBrockbank, JustinNoritz, GareyBrown, MelanieNuss, Henry J.Brown, SamanthaO`Rourke, MargaretBruyneel, LukOkhuysen-Cawley, ReginaBuchowski, MacO'Malley, LucyButler, Marilyn W.Oostenbrink, RianneCabanillas, BeatrizOrtiz, RobinCady, Rhonda G.O'Shea, JoyceCaicedo, CarmenOsier, NicoleCamenga, Deepa R.Owili, PatrickCanbay, AliPacifico, LuciaCanu, WillPallikkuth, SureshCarney, Paul R.Palm, ØyvindCarr, Robert A.Paradies, YinCarter, Brian S.Parkes, AlisonCasanovas, CarmenPatman, ShaneCemeroglu, Ayse PinarPavone, MartinoChadi, NicholasPayne, PeterCheung, Po-YinPease, AnnaChhatwal, JugeshPeltz, AlonChilaka, Cynthia AdakuPendergrast, RobertChirica, MirceaPeraica, MajaChung, Dai H.Pérez-Rodrigo, CarmenCiccone, Marco MatteoPezzella, Angelo ThomasCiciolla, LuciaPfeiffer, BethClemente, Maria GraziaPhillips, FaryaClinch, JacquiPielech, MelissaCohen, Arieh SierraPinheiro, JoaquimCohen, EyalPinquart, MartinConstance, Jonathan E.Pinto Guedes, DartagnanContini, SandroPittenger, JaimeCotton, Robin T.Pokorska-Śpiewak, MariaCristea, IoanaPrabhakaran, SreekalaCrowe, SuzannePratt, Iain S.Cruz, Maria LeticiaPrincipe, Maria Ilaria DelCulbert, Timothy P.Raffone, AntoninoCunningham, MelodyRamadan, Hassan H.Daikeler, ThomasRambaud, JeromeDampier, CarltonRamtekkar, UjjwalDavison, KirstenRathinasabapathy, AnandharajanDe La Vega, RocioRempel, John K.De Schweinitz, Peter AlanRobson, Shannon M.DeJesus, JasmineRollins, NancyDhanekula, Raja KoteswarRomo-Palafox, Maria J.Di Martino, GiuseppeRosen, LarryDick, BruceRossignol, DanielDimopoulos, KonstantinosRössler, JochenDore-Stites, Dawn JeanetteRoutledge, MichaelFenaughty, Andrea M.Rüegger, ChristophFerrara, ChristineRuskin, DanielleFink, Ericka L.Russell, Alexandra C.Fitzpatrick, EmerRusy, Lynn M.Flaxman, AbrahamSanchez-Solis, ManuelFleury, Marie-JoséeSanders, LeeFlexeder, ClaudiaSandler, IrwinFrancois, KatrienSandoval, ClaudioFriedland-Little, Joshua M.Sankarasubramanian, VishwanathFriesen, Myron D.Santoro, NicolaFucho, RaquelSawni, AnjuFurman, LydiaSayón-Orea, CarmenGardner, ReneeSchaefer, MichaelGari, MerceSchafer, Ellen J.Geisler, CorinnaSchechter, NeilGeyer, James D.Schlarb, AngelikaGholami, MaryamSchlueer, BarbaraGiardini, AlessandroSchulze-Neick, IngramGien, JasonSchwantes, ScottGodse, AlokSequeira, SoniaGoldman-Mellor, SidraSerai, Suraj D.Golianu, BrendaSherafat-Kazemzadeh, RoyaGomez, CamiloSherwin, Catherine M.T.Gordon, JohnShindou, HideoGraham, H. KerrShoji, KensukeGreenfield, JohnShonkoff, EleanorGreenough, AnneShroff, ManoharGruber, Kenneth J.Shub, Mitchell D.Guinhouya, Benjamin C.Sibinga, EricaHall, DavidSigmund, ErikHamati, AlineSimon, PatrickHan, Seung JinSimons, LauraHao, LeiSingh, ChanpreetHarding, CeliaSkoro-Sajer, NikaHarris, WilliamSlaughter, JamesHassan, Sherif T.S.Sluss, DorothyHauer, Julie MarieSolah, Vicky A.Hazell, PhilipSoma, CaelanHepping, NicoSouth, AndrewHerbert, AnthonySpetea, MarianaHermos, ChristinaStage, VirginiaHimmelmann, KateStanley, Maria A.Hjern, AndersSterrett, Emma M.Hohwu, LenaStores, GregoryHolmes, GeoffreyStormon, MichaelHosie, AnnmarieSulman, Cecille G.Hudson, Darrell L.Sweeney, BrookeHughes, Jean R.Tang, MinghuaHumphrey, LisaTarini, Beth AnneHumpl, TilmanTaylor, C. BarrIceland, JohnTeicher, Carrie LeeImdad, AamerTelford, Rohan M.Iwamoto, ShotaroThompson, Maxine S.Jaaniste, TiinaThomsen, IsaacJain, AmishThomson, JoannaJaparidze, NatiaTibboel, DickJastrowski, Mano Kristen E.Tiniakou, DinaJegaskanda, SinthujanTokuyama, ShogoJena, PrasantToossi, SaiedJetelina, Katelyn KassarjianTran, Susan T.Job, KathleenTulloh, RobertJones, ChrissieTurner, ElizabethKaiser, PamelaTutor, James D.Kang, Bum-YongVahlkvist, SigneKaplan, Edward LawrenceVajro, PietroKatwa, UmakanthVan Baar, AnneloesKavanagh, Katherine F.Van De Wetering, Marianne DesireeKavanagh, PatriciaVan Der Veek, ShelleyKeenan, MickeyVeatch, Olivia J.Kemper, KathiViala, JeromeKim, Harris Hyun-sooVolkert, ValerieKini, UshaWallace, DustinKirsch, RoxanneWeaver, Meaghann S.Kocken, Paul L.Weiner, Shira SchecterKohen, Daniel P.Weiss, MargaretKolen, Angela M.Weiss, ShellyKoot, Bart G.P.Weydert, JoyKotagal, SureshWhite, Mary L.Kovesi, TomWilsey, MichaelLacy, KatieWitkamp, Frederika EricaLakshminrusimha, SatyanWong, Judith Ju-MingLane, ShellyWright, NicolaLaRovere, Kerri L.Wu, FeitongLavine, JoelWu, Tai-weiLee Hon, WahXu, Feng-LianLightman, SusanYunus, FakirLim, CrystalZadeh, SimaLitt, JohnathanZandieh, Stephanie O.Long, XiZapolski, TamikaLopes, LuisZurynski, Yvonne

